# Dural tear is associated with an increased rate of other perioperative complications in primary lumbar spine surgery for degenerative diseases

**DOI:** 10.1097/MD.0000000000013970

**Published:** 2019-01-04

**Authors:** Shota Takenaka, Takahiro Makino, Yusuke Sakai, Masafumi Kashii, Motoki Iwasaki, Hideki Yoshikawa, Takashi Kaito

**Affiliations:** a Orthopaedic Surgery, Osaka University Graduate School of Medicine, Suita, Osaka; b Orthopaedic Surgery, Toyonaka Municipal Hospital, Toyonaka, Osaka; c Orthopaedic Surgery, Osaka-Rosai Hospital, Sakai, Osaka, Japan.

**Keywords:** complication, delirium, dural tear, durotomy, infection, lumbar spine, multicenter

## Abstract

Prospective case-control study.

This study used a prospective multicenter database to investigate whether dural tear (DT) is associated with an increased rate of other perioperative complications.

Few studies have had sufficient data accuracy and statistical power to evaluate the association between DT and other complications owing to a low incidence of occurrence.

Between 2012 and 2017, 13,188 patients (7174 men and 6014 women) with degenerative lumbar diseases underwent primary lumbar spine surgery. The average age was 64.8 years for men and 68.7 years for women. DT was defined as a tear that was detected intraoperatively. Other investigated intraoperative surgery-related complications were massive hemorrhage (>2 L of blood loss), nerve injury, screw malposition, cage/graft dislocation, surgery performed at the wrong site, and vascular injury. The examined postoperative surgery-related complications were dural leak, surgical-site infection (SSI), postoperative neurological deficit, postoperative hematoma, wound dehiscence, screw/rod failure, and cage/graft failure. Information related to perioperative systemic complications was also collected for cardiovascular diseases, respiratory diseases, renal and urological diseases, cerebrovascular diseases, postoperative delirium, and sepsis.

DTs occurred in 451/13,188 patients (3.4%, the DT group). In the DT group, dural leak was observed in 88 patients. After controlling for the potentially confounding variables of age, sex, primary disease, and type of procedure, the surgery-related complications that were more likely to occur in the DT group than in the non-DT group were SSI (odds ratio [OR] 2.68) and postoperative neurological deficit (OR 3.27). As for perioperative systemic complications, the incidence of postoperative delirium (OR 3.21) was significantly high in the DT group.

This study demonstrated that DT was associated with higher incidences of postoperative SSI, postoperative neurological deficit, and postoperative delirium, in addition to directly DT-related dural leak.

## 1 Introduction

An unintentional dural tear (DT) that occurs during spine surgery for degenerative lumbar diseases is a relatively common complication, with an incidence of 0.2% to 20%.^[1–4]^ Complications that are directly related to DT have been previously reported, including pseudomeningocele, headache, postoperative meningitis, and intracranial hemorrhage.^[5–8]^

The short-term and long-term consequences of DT remain controversial. Some authors have optimistically stated that DT does not have negative impacts on other perioperative complications,^[9–11]^ but the study populations in these studies were too small to evaluate the association between 2 complications that both have a very low incidence of occurrence.^[12]^ In contrast, other authors have concluded that DT increases the occurrence of some perioperative complications, length of stay, and healthcare costs on the basis of large administrative databases.^[13–15]^ However, these administrative databases have inherent inaccuracies introduced by low-quality coding and difficulties in excluding all cases of revision surgery^[16,17]^

The association between DT and other perioperative complications has not been well evaluated for degenerative lumbar diseases. We hypothesized that patients who experience DT during spinal surgery are more likely to have other complications, including intra- and postoperative surgery-related complications and perioperative systemic complications. In the present study, we addressed this question and ensured sufficient statistical power using a registry of prospectively collected multicenter data that originally focused on perioperative complications.

## 2 Patients and methods

All protocols for this prospective study were approved by our institution's review board and ethics committee (No. 11360-3). None of the authors have any conflicts of interest to declare.

### 2.1 Patient demographics

A prospective multicenter analysis of DTs that occurred in a consecutive series of patients with degenerative lumbar spinal surgery between January 2012 and December 2017 was carried out at our 26 affiliated institutions. The surgical strategy used was at the discretion of the spine surgeon. The same questionnaires were sent to participating institutions to collect patient demographic data, surgical information, and data on perioperative complications. Data for each patient were entered right after discharge by the spine surgeons who were responsible for the database at each institution, and the data from 26 institutions were sent to the university hospital for processing every year. The integrity of the data was checked by a data manager at the university hospital. Inconsistencies in the data were corrected through discussions between the data manager and responsible surgeons. Collected patient records and information were anonymized and de-identified before analysis. The database classified the 22356 consecutive spinal surgery cases into 11 categories: primary degenerative cervical spine (N = 4256), primary degenerative thoracic spine (N = 432), primary degenerative lumbar spine (N = 13239), tumor (N = 557), infection (N = 483), osteoporosis (N = 614), dialysis-associated spondylosis (N = 92), deformity (N = 455), revision (N = 1655), and others (N = 285). Of the 13239 patients with a primary degenerative lumbar spine, 13188 were enrolled in this study, after excluding 51 patients with insufficient demographic data. As mentioned above, patients undergoing surgery to correct a deformity and those undergoing revision surgery were excluded. Primary disease was classified into five categories: spinal canal stenosis (N = 6064, 46.0%), degenerative lumbar spondylolisthesis (N = 3767, 28.6%), herniated soft disc (N = 2735, 20.7%), isthmic spondylolisthesis (N = 496, 3.8%), and degenerative scoliosis (Cobb angle ≥30°) without the intention to perform spinal correction (N = 126, 1.0%). Surgical procedures were classified as follows: lumbar interbody fusion, including posterior lumbar interbody fusion or transforaminal lumbar interbody fusion (PLIF or TLIF, N = 5810, 44.1%); decompression only, like laminectomy or laminotomy (N = 5122, 39.8%); discectomy only (N = 2052, 15.6%); and others (N = 204, 1.5%).

### 2.2 Outcomes

DT was defined as a tear that was detected intraoperatively. We evaluated in-hospital surgery-related complications and systemic perioperative complications that were potentially associated with DT. Other intraoperative surgery-related complications that were investigated in this study were massive hemorrhage (>2 L), nerve injury, screw malposition requiring reinsertion, cage/graft dislocation requiring reinsertion, surgery performed at the wrong site, and vascular injury. Postoperative surgery-related complications examined in this study were dural leak, postoperative neurological deficit (a reduction of > 2 grades on manual muscle testing or postoperative sensory disturbance), surgical-site infection (SSI) requiring reoperation, postoperative hematoma requiring reoperation, wound dehiscence, screw/rod failure requiring reoperation, and cage/graft failure requiring reoperation. Information related to in-hospital perioperative systemic complications was also collected for cardiovascular diseases, respiratory diseases, renal and urological diseases, cerebrovascular diseases, postoperative delirium, sepsis, and in-hospital mortality. These systemic complications were defined as conditions in which additional consultation with appropriate specialists was needed. When complications were limited to specific situations (e.g., screw malposition), incidence was calculated using the specific populations (e.g., instrumentation surgery).

### 2.3 Statistical analysis

We used SPSS statistical software version 21.0 (IBM Corp., Armonk, NY) for all statistical analyses. The relationship between DT and other perioperative complications was analyzed. Student *t* test was used to compare the means of continuous variables between the DT and non-DT groups. For categorical values, Fisher exact test was used to evaluate the differences in distributions. DT was analyzed as a risk factor for other complications for which there were statistically significant differences between groups. Odds ratios (ORs) and 95% confidence intervals (CIs) were calculated using multivariable logistic regression models. We sought the best model based on the Akaike information criterion.^[18]^ We controlled for the following potentially confounding factors that were adopted as independent variables: age, sex, primary disease, and type of surgical procedure. Among the surgical procedures, we excluded discectomy in order to avoid multicollinearity, as this procedure was performed only for patients with a herniated soft disc.

## 3 Results

The demographic characteristics of the patients enrolled in the study are shown in Table 1. A total of 451 patients (3.4%, 95% CI 3.1–3.7%) with DT (DT group; 216 men and 235 women; mean age, 69.6 years; range, 19–94 years) were compared with a control group of patients without DT (non-DT group; N = 12737; 6958 men and 5779 women; mean age, 66.7 years; range, 11–94 years). The proportion of female patients in the DT group was significantly higher than that in the non-DT group (52.1% vs 45.4%, *P* = .005). Patients in the DT group were significantly older than those in the non-DT group (69.6 years vs. 66.7 years, mean, *P* <.001). In addition, there were significant differences between groups in disease and procedure distribution. For example, the proportions of lumbar canal stenosis and laminectomy in the DT group were higher than those in the non-DT group (51.2% vs 45.8% and 45.5% vs 38.6%). There were no significant between-groups differences for intraoperative surgery-related complications. In the DT group, dural leak, which is directly associated with DT, was observed in 88 patients (19.5%, 95% CI, 16.0–23.5%). Regarding postoperative surgery-related complications other than dural leak, DT was significantly associated with an increased incidence of SSI (1.8% vs 0.7%, *P* = .015) and postoperative neurological deficit (4.2% vs 1.0%, *P* <.001) compared to that in the non-DT group. Systemic perioperative complications showed similar incidences in both groups except for postoperative delirium (1.3% for the DT group vs 0.4% for the non-DT group, *P* = .011).

**Table 1:**
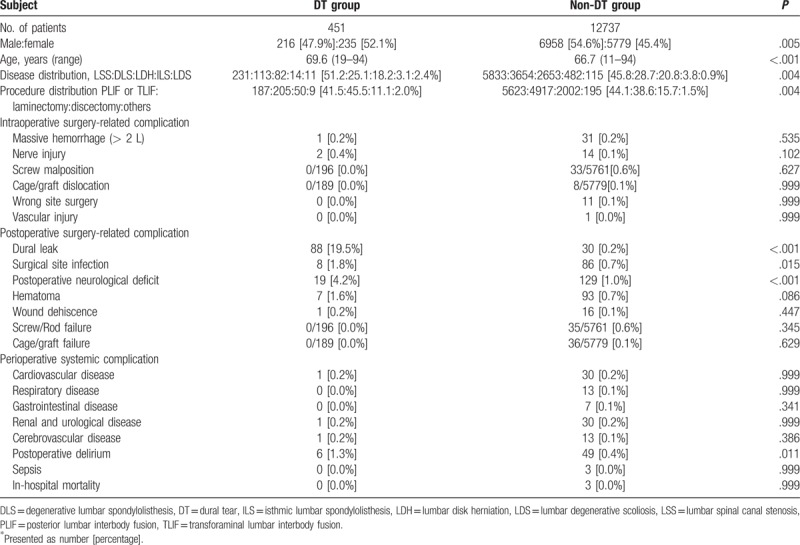
Demographics of patients.

In addition, to calculate the extent of associations between DT and the 4 identified complications, we performed logistic multivariate regression analyses. After controlling for the potentially confounding variables of age, sex, primary disease, and type of procedure, we found that patients with DT had greater odds of experiencing the 4 other complications. Other than dural leak, which is directly related to DT, DT was associated with an increased likelihood of SSI (OR = 2.68, 95% CI, 1.29–5.61, *P* = .009), postoperative neurological deficit (OR = 3.27, 95% CI, 1.55 to 6.89, *P* = .002), and postoperative delirium (OR = 3.21, 95% CI, 1.35 to 7.63, *P* = .008) (Table 2). In the predictive model for postoperative delirium, older age (per decade) was identified as a confounder (OR = 2.86, 95% CI, 1.96 to 4.16, *P* < .001).

**Table 2:**
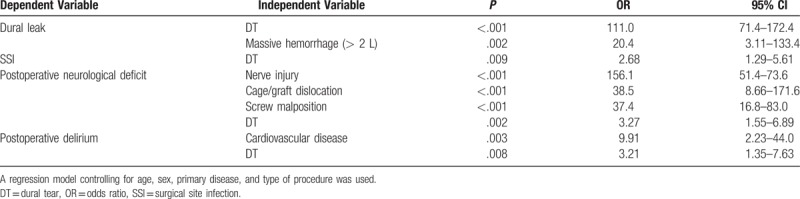
Logistic multivariate regression analyses.

## 4 Discussion

This study demonstrated that DT was associated with a high rate of SSI, postoperative neurological deficit, and postoperative delirium, in addition to the incidence of directly DT-related dural leak.

In the present study, there were statistically significant differences between groups in terms of patient demographics, such as sex, age, disease distribution, and procedure distribution. As with our study, a previous study that investigated DT in spine surgery using a nationwide database demonstrated that the DT group had a significantly higher proportion of female individuals than in the non-DT group (53.6% vs 50.7%, *P* <.001).^[19]^ However, other studies revealed that there were no significant sex differences between groups.^[20,21]^ We do not have a clear explanation for the sex differences. Many studies have shown that patients with DT were older than those without DT.^[13,19–21]^ These results are consistent with our findings. Degenerative changes in the spinal canal, especially in elderly patients, including thicker ligamentum flavum and osteophyte formation, may be one of the reasons for the results.^[22]^ Moreover, the more friable nature of the dura in elderly patients may predispose them to DT.^[23]^ Compared with the non-DT group, the DT group had a higher prevalence of laminectomy and a lower prevalence of PLIF or TLIF. This tendency is consistent with the results of a previous report, according to which laminectomy is a risk factor for DT.^[21]^ During laminectomy, semi-circumferential de-compression must be performed within a more confined spinal canal than in PLIF or TLIF. Under this confined condition, injury to the dura mater may occur during manipulation and nerve retraction.

Adogawa et al assessed the effect of DT on immediate postoperative complications, reporting that DTs occurred in 70 (4%) of the 1741 patients who underwent primary lumbar spine fusion.^[9]^ They concluded that there was no significant difference in the occurrence of SSI (2.85% vs 0.78%, *P* = .32) or postoperative neurological deficit (1.4% vs 0.7%, *P* = .66) between the DT group (N = 70) and the non-DT group (N = 1671). Their results showed trends similar to those in the present study, but they found no statistically significant association between DT and SSI. In particular, their results on the incidence of SSI were stronger than our results (1.8% vs 0.7%, *P* = .015). The difference between their results and ours depended on statistical power. For example, to assure a statistical power of 0.8 to detect group differences, 8450 cases are required to examine the association between a complication (complication A; i.e., DT) with an incidence of 4% and another complication (complication B; i.e., SSI) with an incidence of 3% in the complication A group and of 1% in the non-complication A group. The present study included a sufficient (and not excessive) sample size (N = 13188) for investigating the association between these 2 complications with low incidence.

Puvanesarajah et al reported that the occurrence of DT was significantly associated with a high rate of wound infection in a group of elderly Medicare beneficiaries who underwent primary lumbar discectomy (N = 41655).^[14]^ They found an overall DT rate of 4.9%, with a significant difference in the incidence of SSI between the DT group and the non-DT group (2.4% vs 1.3%; OR 1.88; 95% CI: 1.31–2.70; *P* <.001). Yoshihara et al examined the incidence of DT that occurred during lumbar spinal decompression and lumbar discectomy, risk factors, and patient outcomes using the Nationwide Inpatient Sample and included cases of revision surgery.^[24]^ The incidence of DT was 6.3% (4255/67982) for lumbar spinal decompression, and there was a significant difference in the rates of wound-related complications between the DT group and the non-DT group (3.4% vs. 1.8%, *P* <.001). These findings are comparable with those from the present study. The association between DT and SSI may be explained by 3 factors. First, operative time tends to be longer when an additional procedure is needed to repair DT. Weber et al investigated DT and postoperative cerebrospinal fluid leakage after elective spinal surgery, and they reported that the operative time significantly increased from 116 minutes to 153 minutes when DTs occurred (*P* <.0001).^[25]^ Second, asymptomatic pseudomeningoceles after DT can lead to subcutaneous cerebrospinal fluid leakage accumulation, which can also increase the possibility of SSI due to fistula formation.^[26]^ Third, bedrest, which is needed after patients experience DT, may increase the risk of perioperative complications, including SSI.^[27]^

Previous studies reported results that were comparable to ours, in that DT was associated with an increased risk of postoperative neurological deficit.^[28,29]^ In the present study, DT occurrence was a significant risk factor for postoperative neurological deficit (OR = 3.27, 95% CI = 1.55–6.89). It was also significantly associated with directly related intraoperative complications (nerve injury, cage/graft dislocation, and screw malposition). McMahon et al performed a prospective survey analyzing DTs occurring in 3000 elective spinal surgery cases and reported a significantly higher incidence of postoperative neurological deficit in the DT group than in the control group (7.7% vs 1.5%).^[28]^ Similarly, Williams et al reported a significantly increased incidence of postoperative neurological deficit in the DT group (4.5% vs 1.6%) in their analysis of 108478 patients who underwent spinal surgery. We cannot conclude a causative relationship between DT and postoperative neurological deficit using the results of the present study and previous reports. However, the association between the two complications suggests that neural elements may be injured when the dura is penetrated intraoperatively. In addition, the additional procedures performed by the surgeons to repair DT may lead to the neurological deficit.

Risk factors for postoperative delirium during spine surgery have been investigated in previous studies.^[30,31]^ Postoperative delirium is reportedly observed more frequently in elderly individuals. In the present study, older age (per decade) increased the risk of DT (OR = 2.86, 95% CI, 1.96–4.16, *P* <.001). Interestingly, DT was still a risk factor for postoperative delirium even after adjusting for age, a potentially confounding variable. One study demonstrated an association between DT and postoperative delirium in patients with degenerative spondylolisthesis (OR = 35.8, 95% CI, 1.7–747).^[32]^ This finding is comparable to that in the present study. This tendency may be explained by the requirement for bedrest, which is necessary after DT, but further investigation is needed in this area.

The present study has several limitations. First, our data were limited to in-hospital events. The true incidences of complications may, therefore, have been underestimated. However, the 2014 healthcare data provided by the Organisation for Economic Co-operation and Development showed that the national average length of hospital stay in Japan was 16.9 days, which is much longer than that in the United States (5.5 days) and United Kingdom (6.0 days).^[33]^ These differences are based on differences in the healthcare systems among countries. In Japan, the majority of postoperative complications occur during hospitalization. Second, the database did not include postoperative outcomes related to function, pain, or neurologic status. Third, comorbidities, such as diabetes and smoking status, were not included in this study. These factors may be confounding factors. The results of this study should be carefully interpreted.

In conclusion, this study showed higher incidences of SSI, postoperative neurological deficit, and postoperative delirium in the DT group as well as of directly related complications, such as dural leak. Longer operative time, the need for additional procedures and a longer postoperative bedrest duration could be related to the higher incidence of seemingly unrelated complications in patients with DT.

## Author contributions

**Conceptualization:** Shota Takenaka, Masafumi Kashii, Motoki Iwasaki, Takashi Kaito.

**Data curation:** Shota Takenaka, Takahiro Makino, Yusuke Sakai, Masafumi Kashii.

**Formal analysis:** Shota Takenaka, Takahiro Makino.

**Investigation:** Takahiro Makino, Yusuke Sakai, Masafumi Kashii.

**Methodology:** Shota Takenaka.

**Supervision:** Motoki Iwasaki, Hideki Yoshikawa, Takashi Kaito.

**Writing – original draft:** Shota Takenaka.

**Writing – review & editing:** Takashi Kaito.
